# Abstracts from German Journals

**Published:** 1872-12

**Authors:** Ch. Rauschenberg

**Affiliations:** Atlanta, Georgia


### ABSTRACTS FROM GERMAN JOURNALS.

BY CH. RAUSCHENBERG, M.D., ATLANTA, GEORGIA.

### *WHAT CONSTITUTES THE PROCESS OF TAKING COLD.*

In consideration of the fact that the physiological nature of the so-called process of taking cold, which, in the etiology of diseases, is so often mentioned as their principal cause, has as yet been very imperfectly understood, Professor D. I. Rosenthal, of Erlangen, Germany, has instituted a series of experiments on warm-blooded animals, with a view of defining more clearly the physiological alterations in the organism of animals exposed to sudden changes of temperature.

He has published a small work*, and delivered a lecture on this subject before the German Association for the Cultivation of Public Health, the most important features of which are embraced in this article.

The introductory theoretical points necessary for the understanding of Dr. Rosenthal's investigations are the following:

1. All warm-blooded animals are warmer than the surrounding atmosphere, and therefore normally lose caloric by radiation from the surface of their bodies; hence the surface temperature must always be lower than that of the interior of their bodies.

2. The influence of this loss of caloric by radiation from the surface. The refrigeration extends—in consequence of the distribution of warm blood by the circulation—only to a small depth into the interior from the surface of the body, or, more pointedly, it extends just as far into the interior as the refriger-

---

*Fur Kentniss der Wärmeregulirung bei warmblütigen Thieren Erlangen, 1872

ation by radiation over-balances the equalizing influence of the higher temperature of the circulating blood from the interior of the body.

Hence there is an interior central region of comparatively large extent and of very nearly equal temperature, and between it and the exterior coldest cortical layer of the body, an intermediate zone in which the gradual transitions of the higher temperature of the center to the lower of the surface exist. The rectum of the human body offers ample opportunity for testing the correctness of this statement, as the sigmoid flexure constitutes no obstacle to the careful introduction of the thermometer. The cavity of the mouth, the vagina, the axilla, etc., where such measurements have generally been made, laying within this intermediate zone, show only temperatures approaching that of the center of the body.

The greater the degree of refrigeration or the loss of caloric on the surface, the further will it extend to the interior, and the thicker will the transition zone of gradually rising temperature become, and if the surface loss is sufficiently great, the temperature of the center, unaffected by the usual fluctuations, will be lowered in proportion to the degree of the surface refrigeration, while with a diminished loss of caloric, diminished refrigeration will have the opposite effect.

For the further understanding of the changes which the refrigeration of the surface produces, it is necessary to take into consideration the part which the circulation takes in this process.

The blood, which enters the arteries from the heart, comes from the center of the body, and must have the high degree of temperature of the interior, the surface of the body receives its warmth from it, and the more of this warm blood is contained within the capillaries of the surface, the warmer it will be. The warmer the surface becomes, the more caloric it gives off to the surrounding atmosphere ; therefore, the more the blood-vessels have become dilated, and the larger the amount of blood which in the surface vessels comes in contact with the atmosphere, the greater must necessarily be the loss of caloric of the body.

Hence any cause which produces physically or by diminished or interrupted innervation of the vaso motor nerves, a relaxation of the walls of the blood-vessels, and an increase of their volume must produce a sinking of the temperature of the body, particularly when this dilatation takes place prominently within the vessels of the skin, the locality where this refrigeration is carried on. This sinking of the temperature of the body is observed in animals whose skin has been varnished or extensively scalded.* A dilatation of the capillary vessels of the surface does, also, take place when animals are placed in an atmosphere of a higher grade of temperature, and this dilatation of the capillaries enables the surface of the body to radiate an amount of caloric corresponding to the high temperature of the body, and thus to maintain the bodily temperature at the normal standard, in spite of the small difference between the temperature of the body and the surrounding atmosphere, thereby becoming its regulator.

This regulation of the temperature of the body by the increased radiation of caloric from the turgid surface of the body is, according to Dr. Heyman's experiments, possible, as long as the surrounding atmosphere has not over 32° C.(89.6° F.) temperature. Whenever that temperature rises from 32° to 36° C. (89.6° to 96.8° F.) the body rises from 41° to 42° C. (95.8° to 107.6° F.). Animals can bear this degree of temperature a long time without any danger to their life. They lay exhausted with increased frequency of pulse and respiration, and with enlarged blood-vessels, as can be plainly seen on the capillaries of the ears. Whenever the temperature of the surrounding atmosphere is increased over 36° up to 40° C. (96.8° to 104.0° F.) the temperature of their bodies rises very quickly to from 44° to 45° C. (111.2° to 113.0° F.), the respiration becomes very frequent, the pulse uncountable, the pupils very large, all the muscles become relaxed, and if the animal is allowed to remain any length of time it generally dies. If these animals, after remaining a while in these high temperatures, are brought into a medium one, *this bodily temperature sinks rapidly, not to the normal standard, but considerablg below the same.* It is a frequent occurrence that an animal whose temperature had been increased to from 42° to 44° C. (107.6° to 111.2° F.) shows, on removal to a colder atmosphere suddenly a temperature of 36° C. (96.8° F.), and frequently less, and that it remains that low for hours, and rises only very

*Atlanta Medical and Surgical Journal, August, 1872. Page 289.

slowly to the normal degree.

There can be no doubt that this subsequent decrease of the temperature of the body depends upon a morbid dilatation of the blood-vessels. They have been paralyzed by the high temperature*, and remain so sometime in the lower temperature, therefore the animal must loose more caloric than it would in the normal condition of the vessels at the relative difference of its own and the surrounding temperature. This refrigeration of the body following an abnormal over-heating of the same, is, according to Dr. Heyman, the main constituent of the so-called process of taking cold.

The same occurrence, which was observed by our author on his test animals—the sudden abnormal decrease of the bodily temperature—undoubtedly takes place very frequently in human beings, when, after having been exposed to high temperatures, they are afterwards placed in moderate or cold ones. Temperatures from 30° to 35° C. (86° to 95° F.) frequently exist in ball and other rooms, and men, after leaving them, are often exposed to much lower ones than the animals in the above mentioned experiments. Therefore the same rise above the natural temperature and the same fall below it afterwards, as observed in Dr. Heyman's test animals, must occur in human beings under the same circumstances, particularly when active motion, for instance, dancing, and afterwards the effect of cold air, perhaps in the form of a cold wind, increases the difference of the temperatures between the body and the surrounding atmosphere. The refrigeration within itself does not constitute the process of taking cold in the strictest sense, but the suddenness of the change is the most important factor in producing the deleterious effect.

When the over-heated body with its abnormally dilated surface vessels is suddenly exposed to the cold air, it does not only lose a considerable quantity of caloric at once, but the suddenly refrigerated blood of the surface soon comes in contact with the internal organs and cools them off much more suddenly than it does under the influence of a cold temperature alone without the previous effect of a high one. The general refrigeration of the entire body will, therefore, not only be

*See Atlanta Medical and Surgical Journal, August, 1872, Page 291.

much greater, but much more sudden. This sudden refrigeration can easily cause a morbid condition of any one internal organ, particularly if the same is predisposed to disease from other causes.

The greater the relaxation of the surface vessels the less resistance they will offer to the loss of caloric, and the less they will contract under the influence of a certain degree of low temperature than in the normal state.

This is of importance, not only in relation to its effect upon the skin, but upon other parts of the body. The skin itself may, by this sudden change, become the seat of morbid phenomena (phlegmonous or similar inflammations). In other parts of the body, the contraction of the cutaneous capillaries will, whenever it takes place, produce a collateral hyperæmia which, in connection with the afflux of abnormally refrigerated blood, may induce morbid phenomena elsewhere; although the very relaxation, or as our author is inclined to call it, the paralyzed condition of the surface capillaries will moderate the collateral hyperæmia, and make it a subordinate phenomenon to the great loss of caloric; the sudden refrigeration of the body which always takes place when the over-heated body, with its dilated and engorged capillaries is at once transferred to a colder atmosphere.

From all that has been said, the great importance and practical usefulness of cold effusions, cold baths and shower baths, as a means to counteract a habitual pre-disposition to take cold, must become very evident. Repeated refrigerations of the skin by these means must increase the tone of the capilliaries and habituate them to relax less under the influence of a higher temperature, and therefore to contract sooner to the normal size, when suddenly exposed to a cold one, whereby the extreme degrees of the above described process of taking cold are prevented, and a more normal equilibrium of the bodily temperature maintained under all circumstances.

Daily experience teaches the medical practitioner that people who guard most anxiously against every possible chance of taking cold are most frequently its victims. Geiger* in a paper on the mortality of children at Würtzburg, Germany, shows

*Deutche Vierteljahrschrift fur Gesundheits pflege III., 520.

that disease of the respiratory organs cause, in the first year of life, the death of relatively many more legitimate than illegitimate children; while the contrary is true of diseases of nutrition, proving that the too great care of fond mothers to their offspring frequently produces what it is intended to prevent.—*Berliner Klinische Wochenschrift, No.* 38, 1872.

